# Biochemical Benefits, Diagnosis, and Clinical Risks Evaluation of Kratom

**DOI:** 10.3389/fpsyt.2017.00062

**Published:** 2017-04-24

**Authors:** Dimy Fluyau, Neelambika Revadigar

**Affiliations:** ^1^Brain Health, Emory University, Atlanta, GA, USA; ^2^Columbia University, New York, NY, USA

**Keywords:** *Mitragyna*, *Mitragyna speciosa* extract, kratom, benefits, risks, fatality, toxicity

## Abstract

**Background:**

Kratom (*Mitragyna speciosa*) is a tropical tree with a long history of traditional use in parts of Africa and Southeast Asia. Kratom is also known as Thom, Thang, and Biak. Its leaves and the teas brewed from them have long been used by people in that region to manage pain and opioid withdrawal and to stave off fatigue. Kratom is actually consumed throughout the world for its stimulant effects and as an opioid substitute (in form of tea, chewed, smoked, or ingested in capsules). Some case reports have associated kratom exposure with psychosis, seizures, intrahepatic cholestasis, other medical conditions, and deaths. The clinical manifestations of kratom effects are not well defined and the clinical studies are limited. Data research suggest that both stimulant and sedative dose-dependent effects do exist, in addition to antinociceptive, antidepressant activity, anxiolytic-like effects, and anorectic effects, but a growing concern for the drug’s effects and safety of use has resulted in national and international attention primarily due to an increase in hospital visits and deaths in several countries that are believed to have been caused by extracts of the plant. There is a dearth of double blind controlled studies. In this study, we aim to use existing literature to clarify both benefits and risks of kratom as well as its diagnosis evaluation as kratom misuse is an emerging trend in the Western world.

**Methods:**

Literature review using databases such as Embase, Medline, PubMed, Cochrane Library, and Mendeley from 2007 to 2017 were evaluated by all authors to analyze current state on benefits, risks, and diagnosis evaluation of kratom (*M. speciosa*).

**Results:**

Data analysis suggested that kratom possesses some benefits such as stimulant and sedative effects as wells as antinociceptive effects. It seems to inhibit pro-inflammatory mediator release and vascular permeability and can enhance immunity. In addition, it may be an antidepressant and anorectic. However, kratom can cause intrahepatic cholestasis, seizure, arrhythmia, impair memory function, coma, and death. Psychological manifestations described are euphoria and feeling relaxed to severe symptoms such as aggression, hostility, and psychosis. Medical manifestations described are polyuria, dry mouth, vomiting, and jerky movements. Currently, liquid chromatography/mass spectrometry and ion mobility spectrometry (IMS) are suggested as the most promising to rapidly screen kratom products providing a positive success rate.

**Conclusion:**

Our data analysis has not determined if biochemical benefits of kratom may prove to outweigh its toxicity and risks. On the contrary, it seems that its potential side effects outweigh the benefits, and severe and real health hazards can, insidiously, lead to death. Kratom clinical, psychological, and medical manifestations can be disturbing. Kratom (*M. speciosa*) use, among multiple compounds of the leaf, appear to be increasing in the Western world. Promising methods to accurately identify kratom compounds are still ongoing.

## Introduction

**Figure 1:**
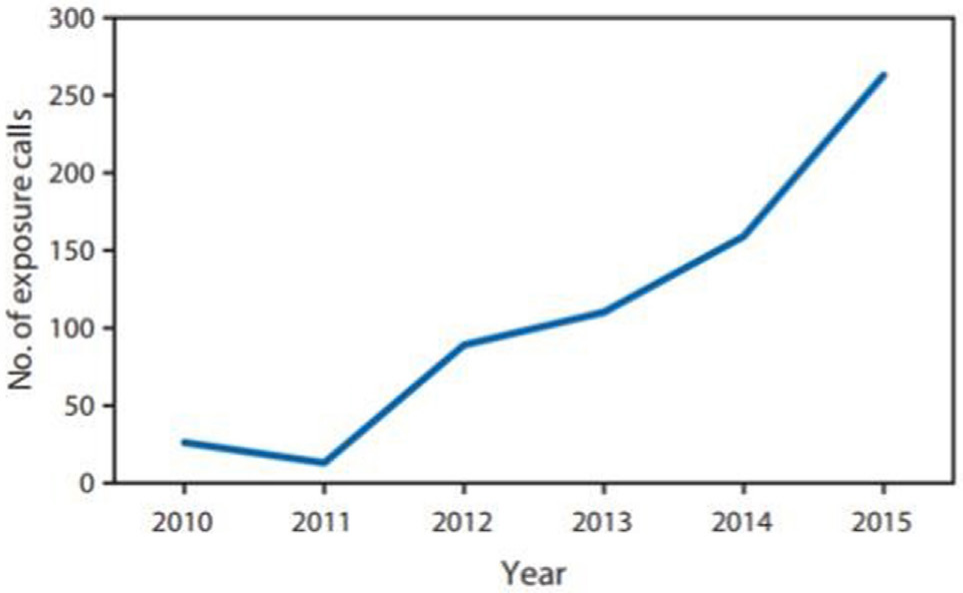
**Number of reported exposure calls to poison centers related to kratom use, by year—National Poison Data System, Unites States and Puerto Rico, January 2010–December 2015**. Copied from MMWR/July 29, 2016/Vol. 65/No. 29 US Department of Health and Human Services/Centers for Disease Control and Prevention.

In the U.S., the Drug Enforcement Administration (DEA) includes kratom on its “Drugs of Concern list,” which means that kratom is not currently regulated by the Controlled Substances Act, but it can pose risks to persons who abuse it. Of note, the National Institute of Drug Abuse has identified kratom as an emerging drug of abuse (1). During 2010–2015, U.S. poison centers received 660 calls about reported exposure to kratom. The number of calls increased 10-fold from 26 in 2010 to 263 in 2015 (1) (Figure 1). Health-care provider reports constituted 496 (75.2%) of calls. Among calls, 487 (73.8%) exposed persons reported intentional exposure and 595 (90.2%) reported ingestion of the drug. Isolated kratom exposure (single exposure) was reported in 428 (64.8%) cases (1). The 2016 report of the U.S. poison centers mentioned one death of a person who took paxil and lamictal in addition of using kratom.

An important note of the report is that among 486 kratom exposures, medical outcomes associated with kratom exposure were minor, and those outcomes were resolved rapidly with no residual disability for about 24.5% exposures, there were some moderate outcomes, non-life threatening, with no residual disability, which required treatment for about 41.7% exposures, and major life-threatening signs or symptoms, with some residual disability for about 7.4% exposures (1). Kratom seems to be a major concern in Florida, USA. On October 8, 2014, CBS Miami reported the death of a 17-year-old young male who used kratom at a kava bar with friends. The authority found his car on the I-95 with six empty packets of kratom and two full ones. Prior his death, the victim’s mother described him as being argumentative and delusional. She added that sometimes the victim’s eyes would roll back in his head. Since then, the Florida Senate introduced a bill in an effort to control kratom or *M. speciosa* as a schedule I substance. The bill was amended to list mitragynine and 7-HMG instead (2). The clinical manifestations of kratom are still not well defined. Published case reports have associated kratom exposure with deaths (3). Research data and surveys suggest that both stimulant and sedative effects exist (2). Close to 1–5 g of raw leaves, a low to moderate dose will produce mild stimulant effects and doses from 5 to 15 g of leaves are claimed to exhibit opioid-type effects (2). Users report that the dominant effects are similar to those of psychostimulant drugs (4). The possible analgesic ability of mitragynine is linked to its interaction with supraspinal μ- and δ-receptors (5), and its possible stimulant activity may be due to the blocking of the stimulation of serotonergic 5-HT2A receptors and to the postsynaptic stimulation of the α-2 adrenergic receptors (6). There is a growing concern for abuse and dependence. According to the FDA and the U.S. DEA, kratom can cause withdrawal symptoms such as hostility, aggression, muscle and bone aches, and jerky limb movements. Case reports of hallucinations, delusion, and confusion were reported by the DEA (7).

## Materials and Methods

**Figure 2:**
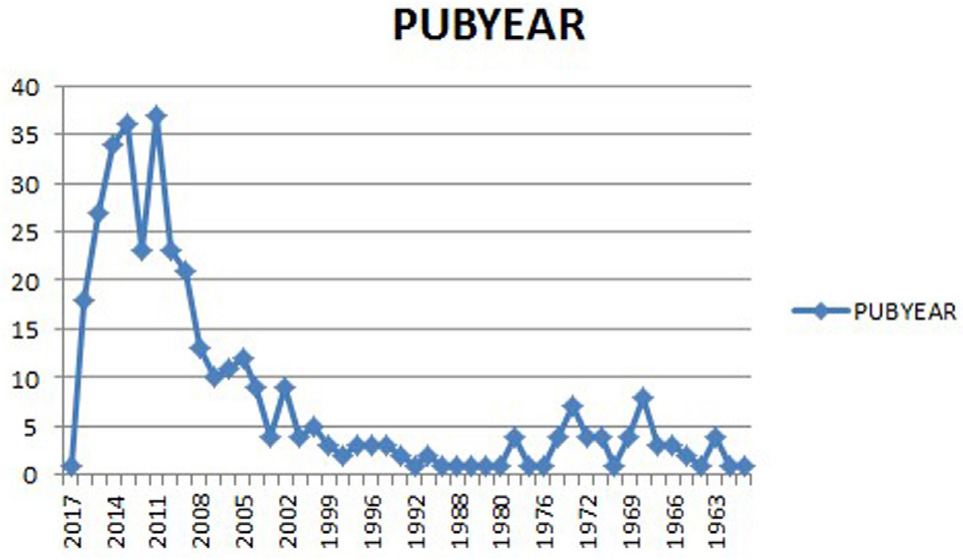
**Kratom publications over the year**. Note the increase of publications from 2009 to 2015, and then a decrease starting around the end of 2015.

We gathered information from databases such as: Embase, Medline, PubMed, Cochrane Library, and Mendeley. Our search terms were kratom, *Mitragyna speciosa, M. speciosa* extract, kratom benefits and risks, *M. speciosa* and toxicity, kratom pharmacology, *M. speciosa* abuse, and kratom deaths. Cochrane library using all search criteria as up to January 2017 (methodology, overview, qualitative, update, diagnostic, etc.) did not provide any result. A total of 195 articles were selected from our search. Our exclusion criteria were *Mitragyna inermis, Mitragyna africanus, Mitragyna parvifolia, Mitragyna rotundifolia, Mitragyna Rubro stipulata, Mitragyna ciliata, Mitragyna stipulosa, Mitragyna tubulosa, Mitragyna hirsuta*, and *Mitragyna savanica*. Our inclusion criteria were *Mitragyna, M. speciosa, M. speciosa* Korth, and kratom. Among the 195 papers, we selected 120 publications from 1950 to 2017 (Figure 2) based on our search criteria above. Those publications comprised animal studies, reviews, case reports, and human studies (Table 1). Our focus was more in depth from 2007 to 2017 because our data collections that we plotted in the graph below show a pick of publications from 2009 to 2015, which becomes decrescendo after 2015. However, we reviewed important papers published in 1975, 1997, 1996, 1998, 2004, 2006, and 2008. Although there is risk of bias because our search was intended to demonstrate mostly risks (toxicity) of kratom (*M. speciosa*) use and to a lesser degree comparing benefits and risks of the leave, we did review kratom biochemical benefits that we describe in the result section where we present the most supported publications representing the most advanced and recent findings of kratom.

**Table 1:** **Study type**.

Non-human	87
Human	73
Animal experiment	35
Animal tissue	17
Animal model	15
Case report	13
*In vitro* study	13
Animal cell	6
Human cell	5
*In vivo* study	3
Interview	2
Phase 2 clinical trial (topic)	2
Qualitative research	2
Questionnaire	2
Clinical article	1
Evidence-based medicine	1
Experimental model	1
Normal human	1
Phase 1 clinical trial (topic)	1
Phase 3 clinical trial (topic)	1
Phase 4 clinical trial (topic)	1
Semi-structured interview	1
Structured interview	1
Structured questionnaire	1

## Results

### Benefits

These data (2) suggest that kratom possesses both stimulant and sedative effects. Kratom has opiate- and cocaine-like effects and has been used as a stimulant by indigenous in the tropical and subtropical regions of Asia as a substitute for opiate users to combat withdrawal symptoms, to treat muscle ache, fatigue (8), and other conditions (9) in addition to be an analgesic, an opiate substitute, an antihypertensive (10), an antidiabetic and anti diarrheal (9), anti-leukemic (5), anorectic (11), and an immunostimulant (12). It is said that at low doses, kratom produces a stimulant effect (13) while at higher doses, it exhibits opioid-like effect (14). The immunostimulant effect reported by Shaik Mossadeq et al. (12) suggested that the extract of kratom inhibits pro-inflammatory mediator release and vascular permeability in combination with immunity enhancement, stimulation of tissue repair, and healing processes by “release and action of a number of hyperalgesic mediators.” Interestingly, the first pharmacokinetic study in humans (a prospective study) happened in Thailand (15) where 10 chronic, healthy users of kratom completed the study and were given a known amount of kratom tea for 7 days. The finding revealed that the pharmacokinetics of mitragynine is linear, which might explain that mitragynine (kratom) might be easily removed from the circulation without invasive methods such as hemodialysis or hemoperfusion in case of toxicity, and the renal excretion of unchanged mitragynine is very low (15). Theoretically, there is a possibility for *M. speciosa* (kratom) to be medically used as a pain killer and as a better opioid substitute in the future (15). In term of receptors interaction (16), *M. speciosa* seems to possess antinociceptive effects *via* supraspinal and -opioid receptors in both *in vivo* and *in vitro* studies (17), and the opioid receptors, neuronal Ca2^+^ channels, the expression of cAMP and CREB protein, and the descending monoaminergic system mediate the pharmacological properties of *M. speciosa* (16). It appears that *M. speciosa* possess antidepressant activity by its significant reduction in corticosterone levels in mice exposed to the forced swim test and tail suspension test (18). Exposure to *M. speciosa* extract for 7 days increased the time spent in open arm of elevated plus maze, indicating the possibility of anxiolytic-like effects of *M. speciosa* extract (19). The antinociceptive effects seem to be the most studied and 7-hydroxymitragynine might have more analgesic activity than morphine in tail-flick and hot-plate tests. The effect of 7-hydroxymitragynine might be inherent to its capacity to penetrate the blood–brain barrier (20). Even though, 7-hydroxymitragynine has been found to have less capacity to cross the blood–brain barrier than *Mitragyna* (20). The lateral hypothalamus direct inhibition might mainly explain the anorectic effects of *Mitragyna* in rats losing weight due to reduction in water and food intake other than affecting the level of cholecystokinin (21), a peptide hormone of the gastrointestinal system which is associated with hunger suppression. Of note, the cholecystokinin level was not affected by the methanolic extract of *M. speciosa*, so the anorectic effect of the plant extract may be attributed to other factors (22). In a paper published in 2016 in the Journal of Medicinal Chemistry (23), detailed in an *in vitro* and *in vivo* study that *M. speciosa* is a potent antinociception devoid of reward or aversion with diminished antinociceptive tolerance, physical dependence, respiratory depression, or gastrointestinal transit inhibition in mouse models (23). In that study, the author labeled *Mitragyna* #3 (mitragynine pseudoindoxyl), which is an oxidative rearrangement product of the corynanthe alkaloid mitragynine. The author established that #3 demonstrated antinociception *via* mu-opioid receptors and not kappa, delta, and/or α2 adrenergic receptors, and in addition, #3 signaling failed to recruit β-arrestin-2 (23). The author suggested that those properties such as failure to recruit β-arrestin-2 and delta antagonism may both be successful in separating antinociception from unwanted side effects (23). Despite those benefits, toxicities have been reported and some of the reports are contradictory. In a study in 1992, a dose of 920 mg/kg administered to dogs did not reveal toxicity; however, a study in 2013 in rats reported a dose of 200 mg had lethal effects (24). In a paper published in 2014, Lu J et al. (25) found that Mitragynine and its analogs may potentiate Torsade de Pointes through inhibition of IKr in human cardiomyocytes, and Harizal (26), in the “Acute toxicity study of the standardized methanolic extract of *M. speciosa* Korth in Rodent” found an increasing in rat blood pressure after an hour of kratom administration, and a highest dose of the standardized methanolic extraction of *M. speciosa* induced acute severe hepatotoxicity and mild nephrotoxicity (26).

### Risks

The lethal dose of Mitragynine is not well defined. Study in rats reported lethal effects of 200 mg/kg total alkaloid extract of *M. speciosa* (27). In a study conducted by professor Suwanlet (28), 30 male and female reported to be addicted to Kratom were selected. The method of study was based on an interview method using a questionnaire of 30 items. One of the participant reported psychological effects in 5–10 min such as feeling happy, strong and active with a intense desire to work. In the same study, participants with prolong use of *M. speciosa* became thin with darken skin especially in their face in cheeks. Interestingly, the study described “an appearance similar to hepatic face” (28), in addition to dry mouth, polyuria, anorexia, weight loss and frequent constipation. In Suwanlert study, withdrawal symptoms from kratom were reported as hostility, aggression, inability to work, muscle and bone pain, and jerky movements of the limbs. Five cases of psychosis were reported " been seen" by the author at the end of 1974. The most striking one was a 55-year-old who experienced delusions and hallucinations in addition of cloudiness of consciousness (28).

### Hepatic Injury

Intrahepatic cholestasis. Harizal et al. (26) in a study published in 2010, a study focused on determining the acute toxicity of *M. speciosa* Korth standardized methanol extract *in vivo* in 4 week-old Sprague–Dawley rats. The animals were administered varied doses of 100, 500, and 1,000 mg/kg of the extract of *Mitragyna*. The result of the study was noticeable for elevation of ALT, AST, albumin, triglycerides, cholesterol and albumin. Histopathologically, the liver showed moderate destruction of polygonal lobules, dilation of sinusoids and hemorrhagic hepatocytes. Later on, in 2011, the finding reported by professor Suwanlet, “an appearance similar to hepatic face” (28), was exemplified by several case reports of liver toxicity after Kratom use. Kapp et al. (29) described a 25-year-old man who ingested kratom for 2 weeks. The man ordered a powdered substance *via* the Internet,^1^ where it was advertised as an additive for foot baths. He ordered two kinds of powder, called Thai Pimp and Malaysian Green. After he used the powdered substance, he described feeling of being relaxed, tired, and loss of appetite, but did not observe any stimulating effects (29). After cessation of the drug, he developed subjective fever and chills on day 2. On day 5 after cessation, he noticed slight abdominal discomfort. On day 8, he developed intense abdominal pain concomitant with brown discoloration of the urine (29). The next day, he developed noticeable jaundice and pruritus. Pathological study identified a drug-induced cholestatic injury, a canalicular cholestasis (29).

### Neurological Injury: Seizure and Coma

In 2010, Nelsen et al. (30) described a case of a 64-year-old male with history of tobacco and alcohol use disorder who exhibited symptom of seizure at home after ingest tea of kratom and *Datura stramonium*. His urine drug screening was positive for cannabinoids, tricyclic antidepressants, and oxycodone. In 2008, Boyer et al. (31) reported a case of a patient who abruptly stopped using hydromorphone and then started self-managing the opioid withdrawal and pain with kratom. He exhibited sign of a tonic–clonic seizure after taking kratom (the herb) and modafinil (a stimulant) (31). However, there has been no finding of seizure-like activities in the most recent animal studies. In 2016, a study carried out by Yusoff et al. (32) found that administration of mitragynine impaired the acquisition, consolidation, and retrieval of memory phases. Suhaimi et al. (16) concluded in his paper, in rodents, that studies have revealed mitragynine and other extracts of the plants can cause cognitive deficit. In fact, Chittrakarn et al. (22) concluded on a paper that mitragynine produced skeletal muscle relaxation, and kratom extract and mitragynine have a direct effect on skeletal muscle by decreasing the muscle twitch. The compound action potential was blocked by high concentrations of kratom extract and mitragynine. Thus, kratom extract has a greater effect at the neuromuscular junction than on the skeletal muscle or somatic nerve (22). Kratom extract decreased muscle contraction on the isolated phrenic nerve-hemidiaphragm preparation causing complete muscle relaxation at a concentration of more than 0.1 mg/mL, and at a highest concentration used (1 mg/mL) caused completely relaxed muscle contraction in a short time period, in about 15 min (22).

### Pulmonary and Kidney Injury

Published in 2015, McIntyre et al. (33) described a case report of a 24-year-old man whose medical history significant for alcohol abuse and depression was found unresponsive in bed. In addition to kratom concentration in the peripheral blood (0.23 mg/L) and central blood (0.19 mg/L), “therapeutic concentrations” of venlafaxine, diphenhydramine, and mirtazapine were also detected together with a negligible ethanol. The postmortem screened positive for kratom. Autopsy revealed pulmonary edema and congestion, and urinary retention (33). Harizal et al., in “acute toxicity study of the standardized methanolic extract of *M. speciosa* Korth in rodent” (26), did not find significant morphological changes in lung and kidney (mild nephrotoxicity) in histopathological results. Although, the highest dose of *Mitragyna* extract induced acute severe hepatotoxicity (26).

### Cardiac Injury

In a 2014 publication, Lu et al. (25) used human induced pluripotent stem cell-derived cardiomyocytes to determine the cardiotoxicity of Kratom and its analogs. They found that Mitragynine (10 mM) significantly prolonged action potential duration and induced arrhythmia. They suggested that Mitragynine and its analogs may potentiate Torsade de Pointes through inhibition of rapid delayed rectifier potassium current (IKr) in human cardiomyocytes (25).

### Thyroid Injury

Sheleg and Collins (34) in “a Coincidence of Addiction to ‘Kratom’ and Severe Primary Hypothyroidism” described a case of 44-year-old man with history of history of alcohol use disorder who took his wife’s Percocet for pain and then used Kratom that he bought from an Internet vendor. After 4 months of “Kratom” use, the patient gained 60 pounds, became lethargic, and developed myxedematous face. A thyroid panel revealed the patient severe primary hypothyroidism (34). The authors suggested that there is a possibility that high dose of mitragynine might reduce the normal response of the thyroid gland to thyroid-stimulating hormone, and *Mitragyna* may have a possible suppressor effect on the function of the thyroid gland (34).

### Cellular Injury

Saidin et al. (35) studied the cellular toxicity of *M. speciosa* Korth on human cell (human neuronal SH-SY5Y cell line lines). The study found cytotoxicity was preceded by cell cycle arrest mainly at G1 and S phase. Although, the study demonstrated that the concentration of *Mitragyna* required to reduce the relative cell number by 50% (IC50) following 24 h treatment of SH-SY5Y cells was 7.5 × 10^−5^ M (35), the author also note a low doses of *Mitragyna* (3 × 10^−7^ M) could stimulate cell proliferation. The evidence remains that Mitragynine can cause cellular toxicity (35).

### Neuromuscular Injury

In 2010, Chittrakarn et al. (36) suggested that kratom extract may interfere with the neuromuscular junction so that the skeletal muscle could not contract and produce muscle relaxation (36). They postulated (Chittrakarn study) that a high concentration of mitragynine 2 mg/mL produced a blockage of the compound nerve action potential; but a complete blockage did not happen. Kratom extract and mitragynine appeared to have a direct effect on skeletal muscle by decreasing the muscle twitch (36).

### Memory Impairment

The object-in-place task is the ability to detect a particular object relative to its location and surrounding objects are examined (37). Apryani et al. (38) administered Mitragynine doses of 5, 10, and 15 mg/kg to male mice in 28 days consecutive days to study the cognitive (working memory effects) effect of Mitragynine. The cognitive effect was studied using object location task and the motor activity in open-field test. The author suggested that chronic administration of mitragynine significantly reduced the discrimination ratio time on object placement task, thus impairs cognitive function (38). Although the hypothesis that chronic administration of opiates can impair working memory function (39) was evoked to tentatively explain the effect of Mitragynine on cognition, the author suggested that further study is needed to determine the molecular mechanism of mitragynine on working and memory process (38).

### Death

In 2014, Karinen et al. (40) described a case report of an accidental poisoning with Mitragynine. The case was a middle-aged man with a dual diagnosis of substance abuse and mental illness found dead in bed (40). The toxicological analyses revealed zopiclone (0.043 mg/L), citalopram (0.36 mg/L), and lamotrigine (5.4 mg/L). Based on the International Association of Forensic Toxicologists, zopiclone lethal level is 0.6 mg/dL, citalopram lethal level is 5.0 mg/L, and lamotrigine lethal level is 50 mg/L. The patient blood specimen revealed Mitragynine (1.06 mg/L) and 7-hydroxymitragynine (0.15 mg/L) (40). In that case, because the concentrations of zopiclone, citalopram, and lamotrigine were within therapeutic concentration ranges, the author assumed Mitragynine to be the main cause of death (40).

## Diagnosis Evaluation

Clinical manifestations, liquid chromatography/mass spectrometry, and ion mobility spectrometry (IMS). Clinical manifestations: the clinical manifestations of kratom effects are not well defined and the clinical studies are limited. Most of the manifestations are drawn from case reports, individual surveys, and Internet forum (28). Clinical manifestations can be divided in positive and negative symptoms (41). In term of positive symptoms, some users report feeling relaxed, energetic, sociable, and euphoric. Other reports suggest that kratom increases sensory perception and users experience no pain (41). The negative symptoms are mostly extrapolated from kratom side effects or withdrawal symptoms that users describe as stomach ache, nausea and vomiting (41), dry mouth, polyuria, anorexia, weight loss and frequent constipation (28), tiredness and a loss of appetite (29), and psychological symptoms such as hostility, aggression, anxiety, depressed mood, dysphoria, moodiness, annoyance, restlessness, visual alterations and unsteadiness (42), low sexual drive, and irritability (43), in addition to some other symptoms reported such as inability to work, muscle and bone pain, and jerky movements of the limbs (28), and cold symptoms such as chills and sneeze. Some users also report symptoms like sweats, dizziness, vomiting, itching, mouth and throat numbness, and sedation (42). Saingam et al. (43) described a cultural syndrome called the “rain panic,” which is of greatest concern for regular kratom users. They describe it as if they feel frozen into the bone with muscle pain, joint aches, cough, sneezing, and trembling (43).

### Qualification and Quantitation of Kratom: Liquid Chromatography/Mass Spectrometry and IMS

There are currently no standard analytical screening techniques for mitragynine and its metabolites following ingestion limiting its detection to more sophisticated techniques like liquid chromatography-mass spectrometry, and more recently, IMS to evidence its use. Liquid chromatography/mass spectrometry: it is a chemical method, free of salts, non-volatile ion as well as non-volatile buffers that can provide information about structures of individual compounds of complex mixtures *via* fragmentation and quantification.^2^ A paper published in the American Journal of Chemistry and Application in 2015, Zhanglei (44) presented a novel method for screening and identification of mitragynine and 7-hydroxymitragynine in human urine by using a high performance liquid chromatography tandem mass spectrometry. Throughout the course of kratom, several methods have been claiming to be more rapid and selective (44). The author claimed this technique to be more rapid and selective, and it can be applied for routine clinical examination and forensic cases. The same author also concluded that further method is needed for accuracy. Noted that prior to that study, several other methods have been studied for the determination of kratom such as capillary electrophoresis, gas chromatography, coupling of mass spectrometry, and other techniques. The author assumed that prior methods were not used in optimal condition to specify kratom as the sole ingredient in specimen submitted, which makes the high performance liquid chromatography tandem mass more selective (44). IMS: recently, in 2016, Fuenffinger et al. (45) proposed IMS as a new method to detect Mitragynine in Kratom products (45). The group reported that 13 of the 15 samples in the sample set contained mitragynine at levels above the IMS detection limit, providing a positive success rate of identification of *Mitragyna* at 100%, and no false positives were obtained. The group claimed IMS as a suitable method for a rapid screening technique for kratom products containing mitragynine (45).

## Discussion

The analysis of data we collected suggested that kratom possesses some biochemical benefits such as stimulant and sedative effects as wells as antinociceptive effects and other benefits. It is also suggested that kratom can cause intrahepatic cholestasis, seizure, arrhythmia, impair memory function, coma, and death. One important finding in our data analysis is the issue of which compound or extract from the leaf possesses vasodilator, antihypertensive, muscle relaxer, diuretic, immunostimulant, and anti-leukemic properties described by Seaton et al. (10) in the Canadian Journal of Chemistry. We noted that the Seaton study was not specific for kratom or *M. speciosa*, but the study focused on the structure of mitraphylline, which is considered an extract alkaloid of the plant *Mitragyna*. In fact, in this study, mitraphylline was isolated from *Mitragyna rubrostipulacea*, but not from *M. speciosa*. The antileukemic property of *Mitragyna* that we reviewed seems to be more anecdotal than being substantiated by the literature. *M. speciosa* requires further study; however, individuals taking large quantities of these opiate-like (35) materials may be at risk (46), especially those who have a high CYP2E1 activity, such as heavy alcohol users (35). In case of liver injury, the liver damage may be due to the presence of contaminant or error in identification or preparation of the herbal product (42). In a 2016 review, Pantano et al. (47) reported that chronic recreational use of kratom has been associated with rare instances of acute liver injury. There has been no suggested evidence on both animal or human studies of the effect of kratom on the thyroid gland. The toxicity of mitragynine in humans is poorly defined (5). In fact, research that evaluates the toxicity of Kratom, mostly done in animals (rats, mice, dogs, etc.) considers the plant to be minimally toxic, and research evaluating its toxic effects on humans is limited (5). Saidin et al. (35) estimated the daily human use of *M. speciosa* is to be about 17 mg, which is about 20 leaves consumption of 20 leaves per day, at this dose, the consumption is said to be regular, and the potential plasma concentration is estimated to be 10^−9^ to 10^−7^ M (35). Fatalities after kratom consumption have been reported to occur in individuals with blood mitragynine concentrations of between 0.45 and 1.0 μM (35), substantially lower than the threshold of toxicity predicted from this *in vitro* report (35). Saidin et al. recommended further study and advised that individual taking large doses of Mitragynine may be at risk of toxicity in association with heavy use of alcohol (35). Prozialeck, in Update on the Pharmacology and Legal Status of Kratom published in the Journal of the American Osteopathic Association in 2016 (48), addressed imminent key issue and limitation in the study of kratom. The author interrogated the evaluation of kratom product, its confirmation, the standardization of a product that contains different mixture of other compounds that can also be active (48). The author also questioned the administration of a plant material to laboratory animals so it would naturally mimic human consumption (48). A major barrier to study kratom is to accurately determine that the street sales is actually composed of kratom compounds and the origin of its extracts accurately describes similarity of structures involved in both benefits and risks mentioned in the prolific literature of Asia. There is different kratom with different biochemical effects, perhaps, based on the region. Kratom can be just a street name chosen by some people selling drugs with no correlation with *M. speciosa* and its extract. It will seem difficult to associate benefits or risk if one has to study the plant in the Western world and especially in the U.S. where the plant is minimally known or studied. It seems difficult to determine the origin of the sale, which might be useful to compare the compounds for what it seems, and how the same compounds might cause multiple side effects or toxicity. Currently, liquid chromatography/mass spectrometry and IMS are suggested as the most promising to rapidly screen kratom products providing a positive success rate. It will be a burden of cost to screen kratom users in the emergency services in the U.S. where the majority of people with substance use disorders crash.

## Conclusion

We could not make a definitive conclusion based on the analysis of data we collected from eminent databases such as Embase, Medline, Pubmed, and Mendeley. Only one article was available from the Journal of the American Medical Association (JAMA). However, there is an increase in non-medical use (street use) of kratom in Western countries as “natural alternative” for self-treatment of opioid withdrawal and pain. Serious toxicity is rare, usually after high dosage. We suggested that kratom (*M. speciosa*) potential side effects might outweigh its benefits. Rare serious risks such as hepatic injury seem to be alarming. In fact, there is animal study, case reports, conference presentations, and surveys reporting hepatic injury. We also noted from our analysis that rare real health hazards can lead to death. Its striking to understand how kratom clinical manifestations, both psychological and medical, can be disturbing. Kratom (*M. speciosa*) abuse, among multiple compounds of the leaf, appears to be increasing in the Western world with an alarming rate where deaths are reported. Kratom (*M. speciosa*), a leaf with multiple complex compounds and from multiple regions of the globe, is not accurately identified and yet clinically defined. Promising methods to accurately identify kratom compounds are still ongoing, and most of the studies were done in Asia. The Western world, the U.S., lies behind those studies while kratom (*M. speciosa*) is on the rise in this continent and seems to be an imminent endemic problem.

## Author Contributions

DF is the main author of the manuscript and NR is the second author of the manuscript.

## Conflict of Interest Statement

The authors declare that the research was conducted in the absence of any commercial or financial relationships that could be construed as a potential conflict of interest.

## Footnotes

^1^www.thai-kratom.de.

^2^http://scs.illinois.edu/massSpec/ion/lcms.php.
